# Supplementary material for: Actuation of microstructures with spin-current volume effect
Source: Commun Eng. 2024 Mar 7;3:42. doi: 10.1038/s44172-024-00187-3 (PMC10955819; doi:10.1038/s44172-024-00187-3)
Supplement: Supplementary file 2 — Supplemental Information [file 44172_2024_187_MOESM2_ESM.pdf]

Supplementary information for Actuation performance of a microstructure with spin-current volume effect

Yi-Te Huang[1], Kenta Suzuki[1], Hiroki Arisawa[2], Takashi Kikkawa[2], Eiji Saitoh[2,3,4,5] and Takahito Ono[1,6]

[1]Departement of Mechanical Systems Engineering, Tohoku University, Sendai 980-8579, Japan
[2]Department of Applied Physics, The University of Tokyo, Tokyo 113-8656, Japan
[3]WPI Advanced Institute for Materials Research, Tohoku University, Sendai 980-8577, Japan
[4]Institute for AI and Beyond, The University of Tokyo, Tokyo 113-8656, Japan.
[5]Advanced Science Research Center, Japan Atomic Energy Agency, Tokai 319-1195, Japan
[6]Micro System Integration Center (μSIC), Tohoku University, Sendai 980-8579, Japan

Supplementary Note 1| The dependence of charge current in NM material on the mechanical vibration

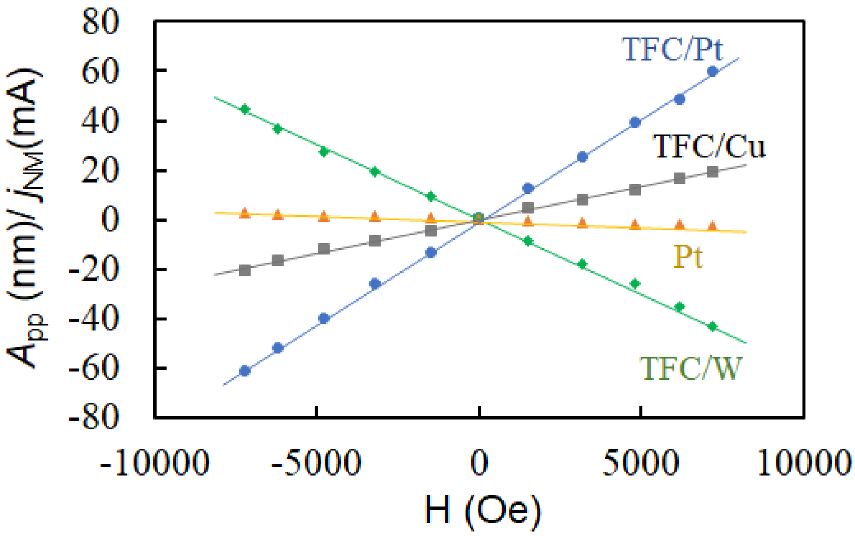

Fig. S1 Magnetic field (H) dependence of the vibration amplitude (A_{pp}) normalized by the charge current flowing in the NM film (j_{NM}) at the resonance for all samples. When the applied total currents are 20 mA, the charge current j_{MN} flowing in the Pt film in TbFeCo(TFC)/Pt, Cu film in TFC/Cu, W film in TFC/W films are estimated to be 15.6, 19.9, and 1.65 mA, respectively. It should be noted that the influence of the Ti adhesion layer is also considered.

Supplementary Note 2| Young's modulus of the composite

In order to obtain the spring constant of the composite diaphragms, the effective Young's modulus E_{eff} of the diaphragms is calculated from the thickness t_n and Young's modulus E_n of each layer, as shown in Table S1. The Young's moduli of Pt, Cu, W, Ti, and polyimide layers are supposed to be 164.6, 115.2, 409.5, 116, and 1.86 GPa [1-5]. The effective Young's modulus is calculated by

$$E_{\mathrm{eff}} = \frac{\sum E_{\mathrm{n}} t_{\mathrm{n}}}{\sum t_{\mathrm{n}}}, \quad \text{(S1)}$$

where n=1, 2, 3, 4, shows each layer's label, t_n is the thickness, and E_n is the Young's modulus.

Table S1. Parameters for composite Young's modulus.

Diaphragm	E_n (GPa)	t_n (nm)	E_{eff} (GPa)
TFC/Pt/Ti/Polyimide	65/164.6/116/1.86	107/100/20/25000	2.86
TFC/Cu/Ti/Polyimide	65/115.2/116/1.86	107/100/5/25000	2.60
TFC/W/Ti/Polyimide	65/409.5/116/1.86	107/100/5/25000	3.77
Pt/Ti/Polyimide	164.6/116/1.86	100/20/25000	2.60

Supplementary Note 3| Power density of the actuation

In order to calculate the power density of SVE, the force is obtained from the displacements and the spring constants of the diaphragm. The residual stress is one of the factor to determine the spring constant. The TFC/Pt, TFC/Cu, TFC/W films on the polyimide plate show a compressive stress of -34, -90, and -26 MPa, respectively. The stress is evaluated by the curvature of the polyimide plate coated with the films [6]. On the other hand, since the polyimide films on which these films are deposited are stretched and bonded to the support substrate with an adhesive layer; therefore, the actual stresses are considered to be low and almost negligible. On the contrary, the reference Pt sample with the polyimide substrate has a tensile stress 50±20 MPa. The spring constant is considered to be significant. Lorentz force can be calculated as $\mathbf{F}_{\text{Lorentz}} = \mathbf{B} \times \mathbf{j_c} L$=0.02 (A) × 0.00157 (m) × 0.72 (T) = 22.61 μN at 20 mA current under 0.72 T magnetic field. Here, L is the effective length of the circular diaphragm with 1 mm radius. This calculated Lorentz force is consistent with the observed value 22.35±8.1 μN. The Lorentz force is generated on all diaphragms; therefore, in order to calculate the actual force by SVE, the correction is needed by the Lorentz force. The parameters for calculation, observed force F_{exp}, corrected force F_{c}, and power density are summarized in Table S2.

Table S2. Various parameters and actuation performance of the diaphragm under 7200 Oe magnetic field with 20 mA charge current. F_{exp} is the experimentally observed force. F_{exp} is the observed force corrected by the Lorentz force.

Sample	Volume (cm^3)	Q-factor	Spring constant (N/m)	F_{exp} (μN)	F_{c} (μN)	Power density (W/m^3)
TFC/Pt	3.36×10^{-7}	19	846	41.10	63.45±8.1	1.17×10^{6}
TFC/Cu	3.36×10^{-7}	36	767	8.27	30.61±8.1	4.07×10^{5}
TFC/W	3.36×10^{-7}	5	1112	-16.24	6.11±8.1	1.09×10^{4}
Pt (Lorentz Force)	3.14×10^{-7}	38	16541	-22.35 ±8.1	-	7.73×10^{4}

Supplementary References

1. Y.C. Huang and T. Ono, "Evaluation of Magnetostriction Properties of Iron Gallium Alloy Thin Films", *Papers of Technical Meeting on Micromachine and Sensor System*, **MSS21-039**, 1-4 (2021-07)
2. Y. Zhou, C. S. Yang, J. A. Chen, G. F. Ding, W. Ding, L. Wang, M. J. Wang, Y. M. Zhang, and T. H. Zhang, "Measurement of Young's modulus and residual stress of copper film electroplated on silicon wafer", *Thin Solid Films*, **460**, 175-180 (2004)
3. J. Merker, D. Lupton, M. Töpfer, and H. Knake, "High temperature mechanical properties of the platinum group metals", *Platinum Metals Rev.*, **45**(2), 74-82 (2001)
4. R. Lowrie, and A. M. Gonas, "Single-Crystal Elastic Properties of Tungsten from 24° to 1800°C", *J. Appl. Phys.*, **38**(11), 4505-4509 (1967)
5. S. Ota, Y. Hibino, D. Bang, H. Awano, T. Kozeki, H. Akamine, T. Fujii, T. Namazu, T. Takenobu, T. Koyama, and D. Chiba, "Strain-induced Reversible Modulation of the Magnetic Anisotropy in Perpendicularly Magnetized Metals Deposited on a Flexible Substrate", *Appl. Phys. Express*, **9**(4), 043004 (2016)
6. Y. T. Huang, and T. Ono, "Magnetostriction and volume magnetostriction of sputtered $Tb_{20}Fe_{24}Co_{56}$ film.", *Material Research Express*, **10** (106101), 1-13 (2023)
